# H2rs: Deducing evolutionary and functionally important residue positions by means of an entropy and similarity based analysis of multiple sequence alignments

**DOI:** 10.1186/1471-2105-15-118

**Published:** 2014-04-27

**Authors:** Jan-Oliver Janda, Ajmal Popal, Jochen Bauer, Markus Busch, Michael Klocke, Wolfgang Spitzer, Jörg Keller, Rainer Merkl

**Affiliations:** 1 Institute of Biophysics and Physical Biochemistry, University of Regensburg, D-93040 Regensburg, Germany; 2 Faculty of Mathematics and Computer Science, University of Hagen, D-58084 Hagen, Germany

## Abstract

**Background:**

The identification of functionally important residue positions is an important task of computational biology. Methods of correlation analysis allow for the identification of pairs of residue positions, whose occupancy is mutually dependent due to constraints imposed by protein structure or function. A common measure assessing these dependencies is the mutual information, which is based on Shannon’s information theory that utilizes probabilities only. Consequently, such approaches do not consider the similarity of residue pairs, which may degrade the algorithm’s performance. One typical algorithm is H2r, which characterizes each individual residue position *k* by the *conn*(*k*)-value, which is the number of significantly correlated pairs it belongs to.

**Results:**

To improve specificity of H2r, we developed a revised algorithm, named H2rs, which is based on the von Neumann entropy (*vNE*). To compute the corresponding mutual information, a matrix **A** is required, which assesses the similarity of residue pairs. We determined **A** by deducing substitution frequencies from contacting residue pairs observed in the homologs of 35 809 proteins, whose structure is known. In analogy to H2r, the enhanced algorithm computes a normalized *conn*(*k*)-value. Within the framework of H2rs, only statistically significant *vNE* values were considered. To decide on significance, the algorithm calculates a *p-*value by performing a randomization test for each individual pair of residue positions. The analysis of a large *in silico* testbed demonstrated that specificity and precision were higher for H2rs than for H2r and two other methods of correlation analysis. The gain in prediction quality is further confirmed by a detailed assessment of five well-studied enzymes. The outcome of H2rs and of a method that predicts contacting residue positions (PSICOV) overlapped only marginally. H2rs can be downloaded from http://www-bioinf.uni-regensburg.de.

**Conclusions:**

Considering substitution frequencies for residue pairs by means of the von Neumann entropy and a *p*-value improved the success rate in identifying important residue positions. The integration of proven statistical concepts and normalization allows for an easier comparison of results obtained with different proteins. Comparing the outcome of the local method H2rs and of the global method PSICOV indicates that such methods supplement each other and have different scopes of application.

## Background

An important objective of molecular biochemistry is a detailed analysis of protein characteristics like functionality, stability, and dynamics. This is a laborious and time consuming task due to the many aspects of protein function and the large spectrum of experimental methods required for their determination. Ideally, one would characterize experimentally the contribution of each individual amino acid residue, which is however not feasible for larger proteins. This is why the biochemical assessment of proteins has to concentrate on a relatively small number of residues. In enzymes, these are the residues directly involved in catalysis and substrate binding; resulting annotations can be found in dedicated databases like PDBsum [1]. However, there are no equivalent databases available when one has to identify residues which are important for stability or other characteristics.

Due to the enormous success of genome sequencing projects, the sequences of more than 17 000 protein families (InterPro Version 45, [2]) are known at date and thus, methods of computational biology are of utmost importance to support their characterization. A large number of *in silico* approaches are at hand to identify important residues. Often, a family-specific multiple sequence alignment (MSA) is the main data source to elucidate the role of the residues; for latest reviews see refs. [3,4]. Most effective is the assessment of residue variation deduced from the corresponding MSA columns. The success of these analyses can be explained with the biochemical properties of the residues: For example, in most cases only one residue-type fulfills all critical requirements at catalytic sites, which prohibits a mutation. Accordingly, a strict residue conservation is a strong indicator signaling functionally important residues [5-8]. In contrast, a prevalent but not exclusively found amino acid is often important for protein stability [9,10], which similarly holds for ligand-binding sites [8]. Interestingly, these less conserved residue positions may bear a pattern indicative of dependencies in the occupancy of two or more positions. The importance of these correlation signals and their consequences have long been realized [11]. Quite different approaches have been introduced to identify correlated residue pairs; see e.g. refs. [12-24]. Unfortunately, these correlation signals, which are due to constraints imposed by the local environment of a residue, can be disturbed by neutral mutations. If an MSA contains sequences from many closely related species, neutral mutations in a predecessor may give rise to a strong correlation signal. Thus, the elimination of highly similar sequences improves the quality of correlation analysis [25,26]. Additionally, other approaches have been proposed to eliminate signals induced by a common evolutionary path of the proteins [27-29].

All these methods for the analysis of correlation patterns are aimed at the identification of pairs of residues, which are functionally or structurally important. More specific methods enable us to predict residue contacts. For the latter application, transitive dependencies, which by definition interlink several pairs of residues, have to be eliminated as well [30]. Different approaches have proven applicable and these algorithms have been named global methods [4]. Among them are PSICOV [31], DCA [32], and EVfold [33]. The common idea of global methods is to treat pairs of residues as mutually dependent entities and to minimize the effects of transitive covariation and phylogenetic noise.

In contrast, most algorithms like those described in refs. [12-24,34] do not correct for transitive dependencies. These approaches have been named local methods [4] as they assume that pairs of residue positions are statistically independent of other pairs. Due to chaining effects, the identified residue positions constituting a pair, can be near to each other or far apart in the protein’s structure.

Most of the local methods rely in one way or another on assessing the mutual information, which is commonly based on Shannon’s entropy [35]. Thus, these local methods deduce a measure for mutual dependencies solely from the amino acid frequencies observed at the positions under study. Consequently, the biochemical properties of the residues are ignored, which may degrade the performance of the algorithm.

One of these local methods is the algorithm H2r [34], which identifies in a first step mutual dependencies between pairs of residue positions and scores in a second step each residue position *k* by the *conn*(*k*)-value, which is the number of significant pairwise correlations it is involved in. Mutagenesis studies with two enzymes demonstrated that positions with high *conn*(*k*)-values have an increased probability of being important for enzyme function or stability [36].

As we were interested to further improve performance of H2r in terms of specificity, we implemented H2rs, which additionally takes into account substitution frequencies for residue pairs. Moreover, H2rs determines a specific *p*-value for each analysis of a residue pair, which facilitates the selection of significant correlation signals. To further standardize the analyses, H2rs normalizes the resulting *conn*(*k*)-values to z-scores, which we named *conz*(*k*)-values. Using a testbed consisting of 200 enzymes, we demonstrated in a comparison with the predecessor algorithm H2r and two alternative algorithms that a larger fraction of residues endowed by H2rs with high *conz*(*k*)-values are located near ligand binding sites. Additionally, we studied in detail the predictions of H2r, H2rs, and the global method PSICOV for five well characterized enzymes. It turned out that the outcome of local and global methods overlapped only marginally and that residues with high *conz*(*k*)-values are functionally or structurally significant.

## Results

### Utilizing the von Neumann entropy to improve the identification of correlated mutations

A classification or regression problem can be solved optimally by means of sophisticated classifiers like support vector machines, given that positive and negative examples are at hand during training. However, there is no clear definition of a correlated mutation. This is why we cannot model the positive cases and can only characterize as precisely as possible the standard situation. Thus, to create a null model, we can deduce mean substitution frequencies for residue pairs from a large number of samples by analyzing known proteins. These substitution frequencies reflect the expected case and will allow us to identify more precisely deviations, which indicate mutual dependencies. Based on this argument, we anticipated an improvement in the identification of correlated mutations, if we additionally take into account the similarity of residue pairs together with their frequencies. Note that frequencies are the only source of information in the standard approach.

The algorithm H2r is based on Shannon’s information theory [35] and computes for each pair of residue positions *k, l* the term *U* (*k, l*) according to

(1) $$U\left(k,l\right)=2\frac{H\left(k\right)+H\left(l\right)-H\left(k,l\right)}{H\left(k\right)+H\left(l\right)}$$

Here, *H*(*k*) is the entropy of an individual column *k*

(2) $$H\left(k\right)=-\sum_{i=1}^{20}p\left(a_{i}^{k}\right)\ln\;p\left(a_{i}^{k}\right)$$

and $p\left(a_{i}^{k}\right)$ is the probability of amino acid *a*_*i*_ at position *k*. The entropy *H(k, l)* of two variables (columns) *k* and *l* is

(3) $$H\left(k,l\right)=-\sum_{i,j}p\left(a_{i}^{k},a_{j}^{l}\right)\ln\;p\left(a_{i}^{k},a_{j}^{l}\right)$$

and $p\left(a_{i}^{k},a_{j}^{l}\right)$ is the probability of the amino acid pair (*a*_*i*_*, a*_*j*_) at positions *k* and *l*. In this context, frequency values deduced from the columns of an MSA served as estimates for probabilities.

Due to normalization, *U*(*k, l*) is a more reliable indicator of co-evolution than a raw mutual information value [14]. As we were interested to improve specificity, we searched for an information theoretical concept allowing the integration of substitution frequencies determined for residue pairs.

The von Neumann entropy (*vNE*) is a generalization of the classical Shannon entropy and has been introduced in quantum statistical mechanics [37]. In computational biology, the *vNE* has been used successfully to characterize the conservation of individual residue positions [38,39]. Extending this concept to residue pairs, we aimed at a novel *U*_*vNE*_(*k*, *l*) term to replace *U*(*k, l*).

The core concept of the *vNE* is the utilization of a so-called density matrix **ρ**_*k*,*l*_, that is, a positive definite matrix whose trace (the sum of the diagonal elements) equals to 1. **ρ**_*k*,*l*_ can be computed for each pair *k*, *l* according to:

(4) $$\rho_{k,l}=P_{k,l}AP_{k,l}$$

Here, $P_{k,l}=\;\mathrm{diag}\left(\sqrt{p_{1}},\ldots ,\sqrt{p_{400}}\right)$ and *p*_1_*…p*_400_ are the pairwise amino acid probabilities $p\left(a_{i}^{k},a_{j}^{l}\right)$ specified in Formula (3). These probabilities satisfy the normalization condition $\sum_{i=1}^{400}p_{i}=1$. **A** is a 400 × 400 matrix that assesses the similarity of residue pairs and it is this matrix that allows us to model substitutions more precisely. If **A** is equal to the identity matrix, then the *vNE* is equal to the Shannon entropy, that is, *vNE*(*k, l*) = *H*(*k, l*); see below. Based on **ρ**_*k*,*l*_, the von Neumann entropy *vNE*(*k, l*) can be calculated as

(5) $$\mathrm{vNE}\left(k,l\right)=\mathrm{vNE}\left(\rho_{k,l}\right)=\;-\sum_{i=1}^{400}\lambda_{i}\log\lambda_{i}$$

by means of the eigenvalues *λ*_*i*_ of **ρ**_*k,l*_. Normalization analogous to Formula (1), which reduces phylogenetic crosstalk, requires corresponding values *vNE*(*k*) and *vNE*(*l*). For their determination, we applied partial traces [40] on **ρ**_*k,l*_ to deduce two density matrices $\rho_{k}^{k,l}$ and $\rho_{l}^{k,l}$, which are specific for a pair of columns *k, l.* The elements of $\rho_{k}^{k,l}$ and $\rho_{l}^{k,l}$ were named *s*_*i,j*_ and *t*_*i,j*_, respectively, and were computed according to

(6) $$s_{i,j}=\overset{20}{\underset{u=1}{\sum \;}}r_{20\left(i-1\right)+u,\;20\left(j-1\right)+u}$$

and

(7) $$t_{i,j}=\overset{20}{\underset{u=1}{\sum \;}}r_{20\left(u-1\right)+i,\;20\left(u-1\right)+j}$$

where *r*_*i,j*_ denotes the appropriate entry in the density matrix **ρ**_*k,l*_. Thus, this approach allows us to deduce all entropy terms from the density matrix **ρ**_*k,l*_, which eliminates normalization problems. We calculate the $\mathrm{vNE}\left(\rho_{m}^{k,l}\right)$ for the residue positions *m* ∈ {*k*, *l*} analogously to equation (5) based on the eigenvalues λ_*i*_ of the 20 × 20 matrix $\rho_{m}^{k,l}$:

(8) $$\mathrm{vNE}\left(\rho_{m}^{k,l}\right)=-\sum_{i=1}^{20}\lambda_{i}\log\lambda_{i}$$

Finally, we define the normalized *U*_*vNE*_(*k*, *l*)-value:

(9) $$U_{\mathrm{vNE}}\left(k,l\right)=\;\frac{\mathrm{vNE}\left(\rho_{k}^{k,l}\right)+\mathrm{vNE}\left(\rho_{l}^{k,l}\right)-\mathrm{vNE}\left(\rho_{k,l}\right)}{\mathrm{vNE}\left(\rho_{k}^{k,l}\right)+\mathrm{vNE}\left(\rho_{l}^{k,l}\right)}$$

Computing these values is straightforward, if a matrix **A** is at hand.

### Computing a matrix A to assess the similarity of residue pairs

**Figure 1:**
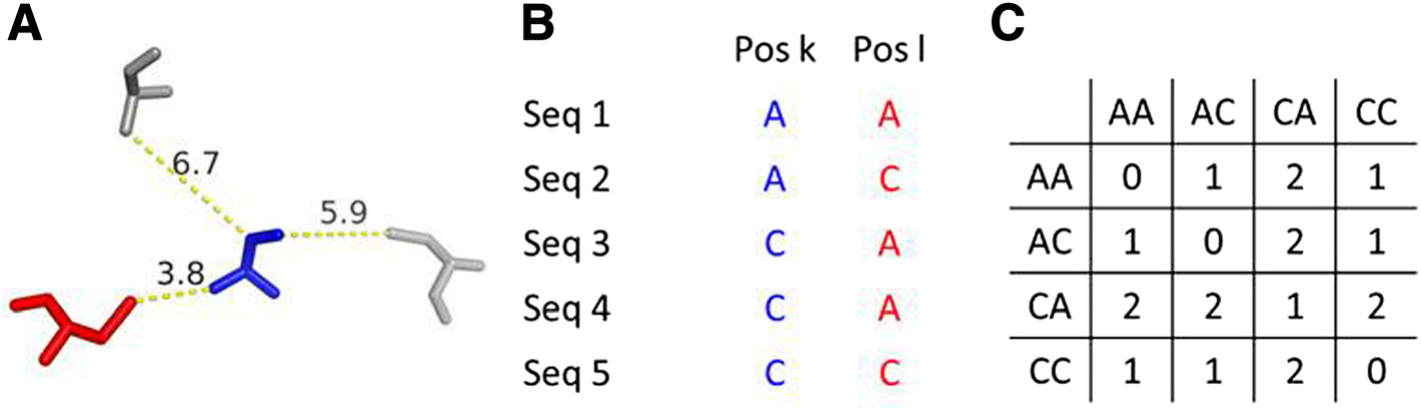
**Computation of a pairwise similarity matrix A. (A)** For each residue (*k*, blue) of our dataset, all neighbors with a distance of at most 5 Å measured between the centers of heavy atoms were determined. Here, it is one residue *l* marked red. **(B)** Residue positions *k, l* were linked with the corresponding columns of the MSA and transition frequencies were deduced from a comparison of the residue pairs. **(C)** In this illustrative example, we observe one transition from AA to AC, two transitions from AA to CA and one transition from AA to CC. Transition frequencies were used to construct the 400 × 400 matrix **A** of substitution frequencies for residue pairs.

In the case of correlated mutations, the matrix **A** is a prerequisite to assess the similarity of residue pairs that occur in homologous proteins at corresponding positions. To determine the 400 × 400 values of **A**, we followed the concept introduced for the BLOSUM approach to score the similarity of amino acid residues based on substitution frequencies [41]. Here, we extended this concept to pairs of residues, as similarly used in P2PMAT [42]. A precompiled and redundancy free set of 35 809 protein 3D structures [43] offered by the PISCES server [44] was used as a representative sample. For each protein, the corresponding MSA was taken from the HSSP database [45] to deduce pairwise substitution frequencies. Based on the 3D structure, those residue pairs *k, l* were identified which contacted each other in the protein. The distances between the centers of any two heavy atoms belonging to one residue each were determined and alternatively the cut-offs 3.5 Å and 5.0 Å were chosen to select contacting pairs. These values correspond to the interval of distances used during CASP9 to identify contacts between residues and ligands [46]. For these cut-offs, we deduced 7 752 286 and 27 283 508 contacts from 15 062 205 sequences, respectively. Then, substitution frequencies were determined by analyzing the corresponding columns of the MSAs; see Figure 1 and Methods. The values of the two corresponding matrices **A**_**3.5**_ and **A**_**5.0**_ were normalized to affirm symmetry. Their comparison indicated highly similar values indicating that this distance is no critical parameter, which is in agreement with findings of CASP9 [46]. As we wanted to consider the larger number of contacts for the determination of the similarity values, we chose **A** = **A**_**5.0**_ for all further computations. This matrix is available as Additional file 1.

### A *p*-value for the strength of correlation signals deduced from a randomization test

Our next goal was to introduce a universally applicable statistical measure for the strength of the pairwise correlations, and we opted for a randomization test. Here, the null hypothesis is that there is no dependency in the pairwise frequencies. Thus, we can assess the strength of each pairwise correlation by shuffling the content of the two columns *k*, *l* under study [47]. As we shuffle the content column-wise, the entropy (conservation) of the two individual columns remains constant; however, we simultaneously degrade the putative correlation between the two residue positions. Then, we can compare the *U*_*vNE*_(*k*, *l*) value deduced from the unaltered combination of residue pairs with a distribution of *U*_*vNE*_(k*, l*) values resulting from many shuffling rounds. Thus, we can rate the correlation strength for this specific combination of residue pairs observed in columns *k* and *l*. Consequently, if the *U*_*vNE*_(k*, l*) values are similarly large or surpass the *U*_*vNE*_(*k*, *l*) value, the correlation is statistically not significant. On the other hand, if all *U*_*vNE*_(k*, l*) values are significantly lower, then this specific *U*_*vNE*_(*k*, *l*) value signals a pronounced dependency in the occupancy of the two residue positions, which indicates correlated mutations.

**Figure 2:**
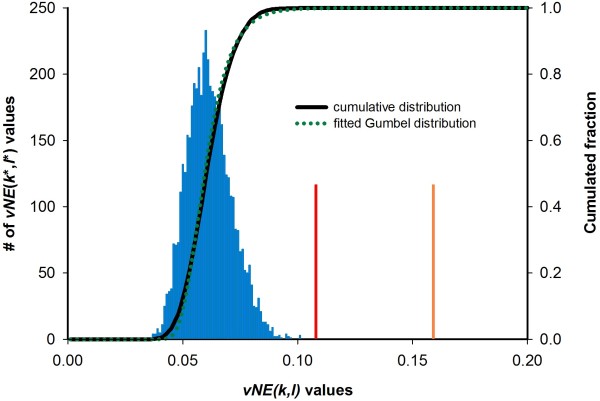
**Distribution of *U***_***vNE***_**() values for one pair of residue positions.** The histogram (blue) shows the distribution of the *U*_*vNE*_(k*, l*) values of the first two residue positions of ssTrpC resulting from shuffling the content of columns *k* and *l* of the MSA. A normality test on this distribution failed (P = 0.991), which indicates that the distribution is not Gaussian. The corresponding cumulative distribution is shown in black. The cumulative *Gumbel* distribution with parameters *μ* and *β* deduced from 25 randomization tests is shown in green. The red line depicts the actual *U*_*vNE*_ value of this pair of residue positions. The orange line shows the *U*_*vNE*_ value this pair would need to surpass a *p-*value of 0.01.

To compute this *p*-value efficiently, the number of randomized samples has to be minimized. Moreover, we need a statistical model which has to be valid, if the number of residue types is relatively small which may cause a skewed distribution. The more conserved the residue positions are, the fewer pairwise frequencies occur and the more the distribution of pairwise frequencies deviates from a normal distribution; compare Figure 2. As we wanted to assess the extremeness of the *U*_*vNE*_(*k*, *l*) values, we selected a Gumbel distribution [48] for modeling. This distribution is specified by only two parameters *μ* and *β* that can be determined in a straightforward manner; see Methods and Formulae 12–14. To confirm that the Gumbel distribution is a proper model, we determined histograms consisting of 1000 *U*_*vNE*_(k*, l*) values each for all of 2 646 726 pairs of residue positions in our dataset. Prior to the computation of the next *U*_*vNE*_(k*, l*) value, columns were shuffled 100*M* times, where *M* is the number of sequences in the respective MSA. A Kolmogorov Smirnov test [49] with α = 0.01 confirmed that the distributions of these *U*_*vNE*_(k*, l*) values and the deduced Gumbel distribution did not differ significantly for 99.14% of all cases. Using the same dataset, we additionally made clear that the two parameters *μ* and *β* can be estimated with adequate precision after 25 instances of randomization. Thus, to compute a specific *p*-value for each residue pair, it is sufficient to compute 25 *U*_*vNE*_(k*, l*) values and to determine one value of the fitted cumulative Gumbel distribution.

For a protein of length *L*, we apply this test *N* = *L*(*L* + 1)/2 times, which suggests to introduce the Bonferroni correction [50] in order to reduce the number of false positive results caused by the frequent application of the test. Thus, a corrected cut-off *c_o* for the corresponding *p*-value *p* is

(10) $$c\_o\left(k,l\right)=\mu -\beta \log\left(\log\left(\frac{1}{1-p/N}\right)\right)\text{.}$$

*c_o*(*k*, *l*) allows for a statistically meaningful and content specific selection of correlated residue positions. *μ* and *β* are defined by Formulae (13) and (14); see Methods.

For the identification of correlated mutations, a *p*-value *p* has to be selected beforehand. Then, all pairs of residue positions with *U*_*vNE*_(*k, l*) ≥ *c_o*(*k, l*) are utilized to compute *conn*(*k*)-values by counting the number of significantly correlated pairs *k* (or analogously *l*) is part of. To further alleviate the comparison of different test sets, *conn*(*k*)-values were transformed to z-scores *conz*(*k*); see Formula (15).

### An *in silico* testbed for the assessment of correlation methods

The ultimate validation of a correlated mutation is a biochemical experiment, which is frequently based on the replacement of residues by the standard amino acid alanine. However, the detailed experimental analysis of a large number of mutations introduced in one protein like dihydrofolate reductase [51,52] is still the exception. This lack of reliable results impedes establishing a *bona fide* testbed for correlation methods and enforces the use of *in silico* surrogates. It is known that many correlated mutations are in close proximity to functional sites [19,47,53-55]. Thus, a testbed has been created that consists of 44 enzymes whose structure and active site residues are well characterized [54]. To assess the quality of correlation analysis, residue positions around functional sites have been counted as positives and all others as negatives [54]. To broaden the statistical basis, we compiled a non-redundant dataset of 200 enzymes, whose functional sites, i.e. catalytic and binding sites, are known and which are represented by a PDB structure and a corresponding MSA in the HSSP database; see Materials. To determine performance values, 64 575 residues were classified and the distances between van der Waals spheres were determined. We regarded all 6192 residues with a maximal distance of 1 Å to a functional site as positive cases and all other 58 383 residues as negative cases. The classification and the resulting performance depends on the chosen *p*-value and the cut-off for *conz*(*k*). This is why we tested several combinations and summarized results in Table 1. For a *p*-value between 10^-2^ and 10^-4^ and a *conz(k)-*threshold of 2.0, the specificity was between 0.97 and 0.98 and precision was between 0.18 and 0.19. For the *p*-value 10^-2^ and the *conz*(*k*)*-*threshold of 4.0, specificity was 1.0 and precision 0.30. For *p*-values ≤ 10^-5^ and *conz*(*k*) = 2.0 the performance reached a plateau. The comparison with the predecessor algorithm H2r made clear that the novel algorithm performed better: Specificity and precision were up to 3% higher. Additionally, we analyzed the same dataset with the algorithms CMAT [56] and SCA [16], which predict pairs of correlated residue positions. Standalone versions as of February 2014 were downloaded and applying the same criteria as above, performance was determined. CMAT was used with default parameters. For SCA, we selected three cut-off values 0.7, 1.5, and 3.0. Performance values were added to Table 1. CMAT reached a specificity of 0.77 and a precision of 0.13. For SCA, the specificity increased from 0.53 to 0.99, and the precision from 0.12 to 0.15, for the cut-offs 0.7 and 3.0. These results indicate that residue positions predicted by H2rs are more likely close to functional sites. Moreover, the number of false positives is lower, as indicated by the higher precision values determined for H2rs. These numbers are a rough estimate of the algorithm’s performance due to the limitations of the *in silico* testbed. However, all other alternative methods of performance evaluation [57] are not applicable here: These are the analysis of simulated MSAs, the determination of the residues’ spatial distance or an assessment of free energy differences derived from double mutants.

**Table 1:** **Performance of four local methods deduced from an *in silico *** **testbed**

	**Cut-off**	**z-score**	**Specificity**	**Precision**
	10^-2^	4.0	1.00	0.30
	10^-2^	2.0	0.97	0.18
	10^-3^	2.0	0.97	0.18
**H2rs**	10^-4^	2.0	0.98	0.19
	10^-5^	2.0	0.98	0.18
	10^-10^	2.0	0.98	0.17
	10^-11^	2.0	0.98	0.17
**H2r**			0.95	0.17
**CMAT**			0.77	0.13
**SCA**	0.7		0.53	0.12
	1.5		0.84	0.15
	3.0		0.99	0.15

For all programs, specificity and precision were deduced from the analysis of 200 enzymes with known catalytic and binding sites. Residues with a maximal distance of 1 Å to a functional site were regarded as positives. All other residues were regarded as negatives. H2r and CMAT were used with default settings. For H2rs, the cut-off was applied to the *p*-value. For SCA, three cut-off values were chosen.

### An assessment of predicted coevolving residues in well-characterized enzymes

To evaluate performance of our algorithm in more detail, we analyzed the H2rs predictions for five well studied enzymes: three enzymes from tryptophan biosynthesis, named TrpA, TrpB, TrpC, dihydrofolate reductase (DHFR), and hexokinase (HK). TrpA and TrpB constitute the heteromeric tryptophan synthase complex, which catalyzes the final reaction of indole-3-glycerole phosphate and serine to tryptophan. TrpA cleaves indole-3-glycerol phosphate to glyceraldehyde-3-phosphate and indole, which is transported through a hydrophobic tunnel to the active center of TrpB. There, tryptophan is synthesized from serine and indole [58]. For the localization of predicted residue positions, we utilized the 3D dataset with PDB ID 1KFC, which is the TrpA/TrpB complex from *Salmonella typhimurium* (stTrpA, stTrpB). The enzyme indole-3-glycerol phosphate synthase (TrpC) catalyzes the ring closure of an N-alkylated anthranilate to a 3-alkyl indole derivative, which is the fourth step in the tryptophan biosynthesis. It adopts the widespread (βα)_8_-barrel fold and has been studied in detail [59]. Here, we utilized the structure of TrpC from *Sulfolobus solfataricus* (ssTrpC, PDB ID 1A53)*.* DHFR catalyzes the reduction of dihydrofolate to tetrahydrofolate via hydride transfer from NADPH. It has been found in most organisms and plays a critical role for cell proliferation and cell growth [60]. We utilized the structure determined for DHFR from *Escherichia coli* (ecDHFR, PDB ID 7DFR). The hexokinase from *Schistosoma mansoni* (smHK, PDB ID 1BDG) is the first enzyme in the glycolytic pathway and catalyzes the transfer of a phosphoryl group to alpha-6-glucose (GLC). The 3D crystal structure contains SO_4_ anions in the catalytic cleft [61]. smHK is the only enzyme of a larger set that has been analyzed previously by correlation analysis and for which the MSA (*smHK_CMA*) was available online. To generate *smHK_CMA,* the authors have used a sophisticated protocol to merge several structure based MSAs [19].

Although local and global methods of correlation analysis have different objectives, we were interested to determine the overlap of their predictions. This is why we also compared the outcome of H2rs and PSICOV [31], which is a global method predicting residue contacts. For PSICOV we analyzed the top *L*/5 predictions, which is the recommended default for a protein sequence of length *L*. An MSA was created for each enzyme by using DELTA-BLAST [62] with the options max target threshold 2000 and expect threshold 10^-10^. The resulting sequences were realigned by means of MAFFT [63] in linsi mode. We were interested in an assessment of the most specific H2rs predictions. This is why we chose the low cut-off 10^-11^ for the *p*-value and a *conz*(*k*)*-*threshold of 2.0. To allow for a comparison, we also listed the *conz*(*k*)-values for all residues predicted by H2r in Table 2. Residues were regarded as functionally important, if they were close to a functional site specified in PDBsum [1]. Thus, all direct neighbors in the sequence were chosen and all residues with a 3D distance of maximally 5 Å (determined between heavy atoms).

**Table 2:** Annotation of residue positions predicted in five enzymes as being important by H2rs and H2r

**Protein**	**Residue**	**H2rs**	**H2r**	**PSICOV**	**Residue’s role**
stTrpA	L100	2.2	3.2	1	Near binding site
	S125	1.1	6.8	1	Stabilizes the active site
	L127	2.0		2	Near binding site
	A129	1.9	5.7	5	Near active site
	I153	0.9	4.6	1	Near active site
	L162	0.7	6.1	0	TrpA/TrpB interface
stTrpB	P7	1.3	6.8	0	ND
	C62	2.2	7.3	0	ND
	G83	1.8	7.2	2	Near binding site
	T88	2.4		1	Near binding site
	Q90	2.4	7.5	0	Near binding site
	V91	2.1		0	Near binding site
	L121	1.8	6.3	1	ND
	C170	4.5		4	End of substrate tunnel
	T190	2.2		6	Metal binding site
	P257	2.2	6.7	0	Near metal ion
	G268	2.3		0	Coordination of ion binding
	F280	2.4	2.8	0	End of substrate tunnel
	M282	2.6		4	Near binding site
	S297	4.2		3	Near metal ion
	F306	-0.8	5.0	0	Metal binding site
	S308	2.4	8.5	0	Metal binding site
	Q312	2.9		0	ND
ssTrpC	I48	2.4		3	ND
	A50	1.4	6.1	1	Near active site
	Y76	1.1	4.0	1	ND
	M109	1.9	4.3	2	Near active site
	I133	2.6	9.8	3	Catalytically important
	V134	2.3		2	Near active site
	I136	2.1		1	ND
	L142	2.7		1	Catalytically important
	N161	1.4	6.9	2	Near active site
	L187	1.8	4.6	1	Mutation L187A is neutral
	A209	2.1		3	Near binding site
	S234	2.1	9.5	4	Phosphate binding site
ecDHFR	A9	2.2		2	Near active site
	W30	2.3		0	Binding site
	K32	2.3		0	Binding site
	M92	3.4		0	Near active site
	G121	2.7	2.8	0	Near active site
	D144	1.9	5.1	0	ND
	H149	2.1	4.4	0	Coupled motion
smHK	T69	2.8		1	Domain interface
	A215	2.6		2	End of domain 1
	C217	2.7	13.9	0	End of domain 1
	A218	2.3		0	End of domain 1
	C224	2.2		0	Begin of domain 2
	V230	2.1		3	Near binding site
	V256	2.1		2	Domain interface
	K290	2.2		0	Near binding site
	D367	1.5	9.8	2	ND
	T409	2.4		1	Near C224
	V412	2.0		0	Near binding site

For the enzymes stTrpA, stTrpB, ssTrpC, ecDHFR, and smHK, H2r and H2r were used to identify important residue positions. For these residues, annotation was deduced from literature. The first column lists the name of the enzyme. The second column gives the residue and its position. The third column gives the *conz*(*k*)-value deduced by H2rs from all *U*_*vNE*_()-values based on a *p*-value of 10^-11^. The column H2r lists mean *conn*(*k*)-values resulting from 25 randomization tests. The column PSICOV lists the number of contacting pairs the residue belonged to. The last column lists the role of the residues, for details see Results. “ND” indicates that we did not find clues to the function of this residue.

**Figure 3:**
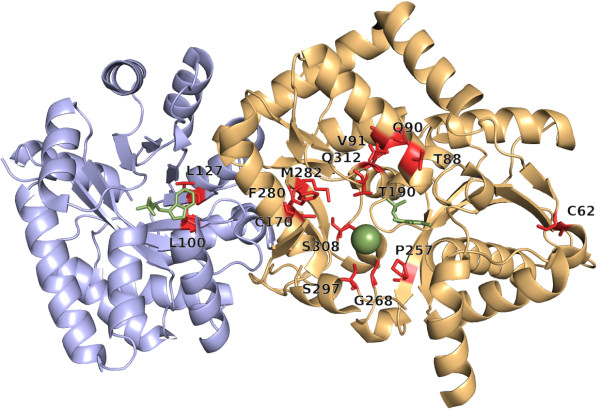
**Residues of the stTrpA/stTrpB complex possessing highest *conz*(*k*)*-*values.** For stTrpA (light blue) and stTrpB (gold), residues with *conz*(*k*)*-*values ≥ 2.0 and *p-*values ≤ 10^-11^ are plotted in red as sticks. H2rs predicted for stTrpA 2, and for stTrpB 13 important residue positions. Ligands indole-3-glycerol phosphate and pyridoxal phosphate are plotted as green sticks. The sodium ion is shown as a green ball.

stTrpA consists of 268 residues, and H2rs predicted two important residues, namely L100 and L127. Both residues are in close proximity to the substrate; see Figure 3. H2r predicted L100, S125, A129, I153 and L162. S125 stabilizes the inactive conformation of the active center [64]. A129 and I153 are near the active site and L162 belongs to the TrpA/TrpB interface [1]. L100 and L127 also belong to the 80 *L*/5 predictions of PSICOV; see Table 2.

For stTrpB, H2rs predicted 13 of the 397 residues as being important; see Figure 3. T88, Q90, and V91 are in close proximity to the substrate binding residue K87 [65]. C170 and F280 are located at the end of the hydrophobic tunnel [66] and T190 and S308 are metal binding sites [1]. G268 is important for the coordination of ion binding [67], and S297 and P257 are in close proximity to the bound sodium ion. M282 is in contact with F280 and S308; see above. The role of the two residues C62 and Q312 is unknown to us. In contrast, H2r predicted five of these residues, namely C62, Q90, P257, F280, S308, and additionally P7, G83, L121, and F306. F306 is a metal binding site, G83 is near the binding site for the substrate and the function of P7 and L121 is unknown to us. Of the 13 H2rs predictions, 5 belong to the 80 *L*/5 contacting residues predicted by PSICOV; see Table 2.

**Figure 4:**
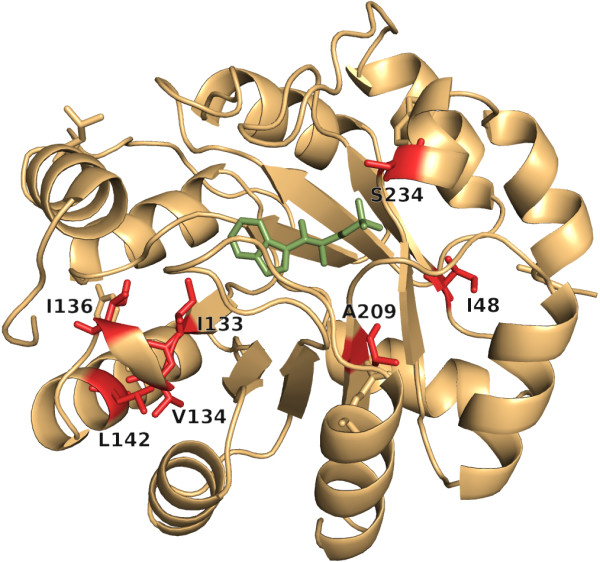
**Residues of ssTrpC with highest *conz*(*k*)*-*values.** For ssTrpC, H2rs identified 7 residues with *conz*(*k*)*-*values ≥ 2.0 and *p-*values ≤ 10^-11^, which are shown as red sticks. The ligand indole-3-glycerol phosphate is shown as green sticks.

For ssTrpC, H2rs predicted 7 important positions; see Figure 4. V134 is near the active site. I133 and L142 are catalytically important: After replacing each of these two residues by alanine, the activity of TrpC dropped 30-fold [68]. A209 is located next to the substrate binding site E210 and the catalytic residue S211 [1]; S234 is known to be a phosphate binding site [1]. The role of the two residues I48 and I136 is unknown to us. H2r detected the phosphate binding site S234, the catalytically important residue I133, plus the residues A50, Y76, M109, N161, and L187. A50, M109, and N161 are near the active site. The role of L187 is unknown however, the L187A mutation has no drastic effect on function and stability [36]. The function of Y76 is unknown to us. All of the residue positions predicted by H2rs belonged to the 50 *L*/5 contacting residue pairs predicted by PSICOV; see Table 2.

**Figure 5:**
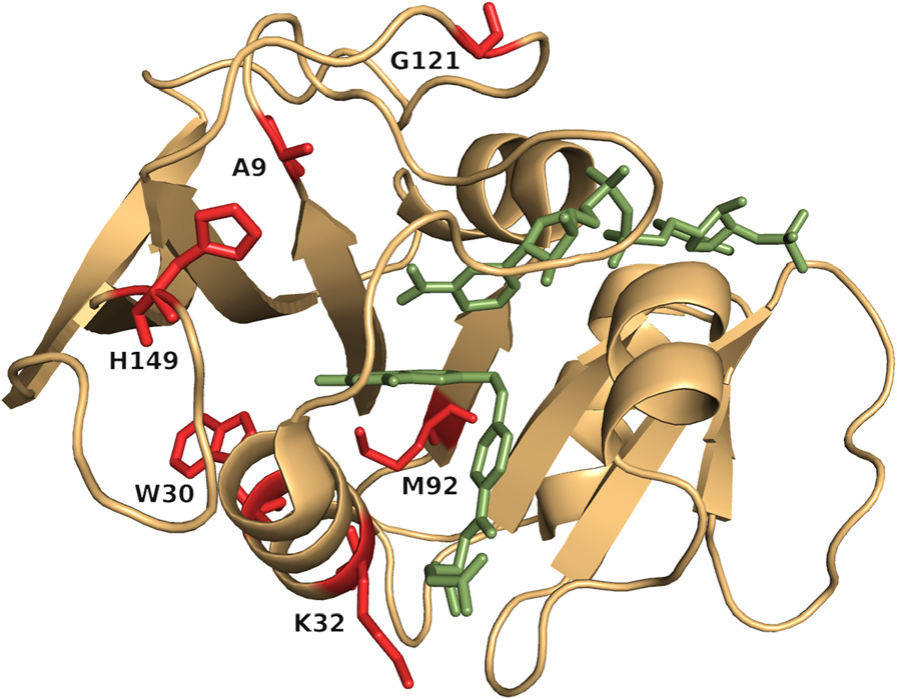
**ecDHFR residues with highest *conz*(*k*)*-*values.** For ecDHFR, H2rs predicted 6 residues with *conz*(*k*)*-*values ≥ 2.0 and *p-*values ≤ 10^-11^, which are shown as red sticks. The ligands folic acid and NADP are shown as green sticks.

For ecDHFR, H2rs predicted six important residue positions; see Figure 5. W30 and K32 are contacting the substrate, whereas A9 and M92 are in close proximity to the binding site A7 and the catalytic site I94, respectively [1]. H149 plays a significant role in the network of coupled motions required for a hydride transfer [69] and a mutation of G121, which lies in proximity of NADPH, reduced the hydride transfer rate [70]. The predecessor algorithm, H2r, identified G121, H149, plus D144, whose function is unknown to us. Of the above sites, only A9 was an element of the 32 *L*/5 predictions of PSICOV; see Table 2.

**Figure 6:**
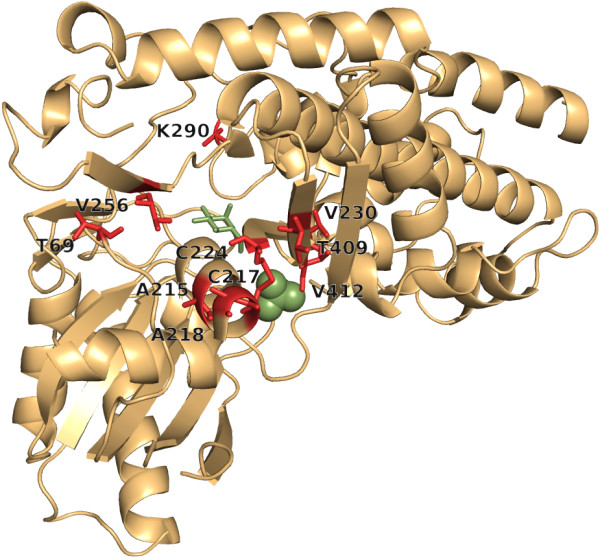
**smHK residues with highest *conz*(*k*)*-*values.** For smHK, H2rs predicted 10 residues with *conz*(*k*)*-*values ≥ 2.0 and *p-*values ≤ 10^-11^, which are shown as red sticks. The ligand GLC is shown as green sticks and the SO_4_ ion in the catalytic cleft as green balls.

smHK consists of a HK type-1 (residues 18 – 218) and a HK type-2 domain (residues 221 – 457); see entry Q26609 of Uniprot [71]. H2rs identified 10 suspicious residues (Figure 6), which we number according to the PDBsum [1] entry 1BDG. A215, C217, and A218 are located at the very end of domain 1, whereas C224 occurs at the very beginning of domain 2 and these four residues are flanking a ß-turn [1]. K290 is a neighbor of Q291 that binds GLC, V230 is a neighbor of I229 (binds GLC) and of T232 (binds SO_4_) [1]. V412 is a neighbor of G414 and S415 that both bind SO_4_[1]. T409 is close to C224 (see above). Only for two residues, namely T69 and V256, their role is unknown to us; however both residues are located at the domain interface at a distance of not more than 5.2 Å. H2r found C217 and additionally D376, whose function is unknown to us. 5 of the H2rs predictions were in the 91 *L*/5 predictions of PSICOV. When utilizing the MSA *smHK_CMA*, H2rs predicted only three residues with a positive *conz*(*k*)-value, which is given in brackets: K295 (3.0), T172 (0.71), and C217 (0.71). T172 binds GLC, and K295 is located next to the GLC binding E294 [1]. For C217, see above. Interestingly, in the 668 sequences remaining in the MSA after filtering, residue positions 217 and 224 were occupied in not more than 43% by cysteines, which form a disulfide bridge that stiffens the orientation of the two domains [1]. Alternatively, the following residue pairs were observed with more than 2% frequency: ST (12.7%), GV (7.8%), SM (6.1%), RT (5.1%), HP (2.7%), AV (2.4%) and RA (2.1%). These distinct pairwise combinations support nicely the idea of mutual dependencies and pairwise correlations.

Although the number of cases is small, these well characterized proteins allow for a more realistic assessment of the prediction performance. Altogether, H2rs predicted 38 important residues and H2r 26, respectively. False positives were 4 (11%) in the case of H2rs and 6 (23%) in the case of H2r. Thus, the resulting precision is 0.89 for H2rs and 0.77 for H2r. These results emphasize the relatively high specificity reached by computing *conn*(*k*)-values and additionally suggest a considerable improvement for the novel algorithm.

## Discussion

### H2rs is a major improvement over H2r

For all well-characterized enzymes studied in Results, H2rs predicted a larger number and a higher fraction of residue positions for which we could rationalize an important role in function or stability. Here, we concentrated on the analysis of residues with a *conz*(*k*)-value ≥ 2.0. Generally, this detailed analysis of five enzymes signals more precisely than the assessment of our *in silico* testbed the improved specificity of H2rs. It was achieved *i*) by replacing Shannon’s entropy by the von Neumann entropy and *ii*) by integrating a more sensitive statistical approach that adapts to the composition of each pair of MSA columns. Based on this dataset, we can expect a 10% increase in specificity to nearly 90%. However, this improvement has to be paid with a much longer execution time: Computing the von Neumann entropy requires the determination of eigenvalues, which is time-consuming and the determination of *p*-values further increases the execution time by a factor of 25. One way of accelerating the calculation of entropy values might be an application of the Rényi entropy [72], which is a generalization of the von Neumann entropy.

For 0 < α ≠ 1, the α-Rényi entropy is given by $\alpha -\mathrm{RE}\left(k,l\right)=\frac{1}{1-\alpha }\log\sum_{i=1}^{400}\lambda_{i}^{\alpha }$ and for α → 1, we recover the Neumann entropy *vNE*(*k, l*). Interestingly, for α = 2, the calculation of the α-Rényi entropy does not require the eigenvalues of the matrix **ρ**_*k,l*_ but only the diagonal entries of the square of **ρ**_*k,l*_, which drastically speeds up the computation. However, it has not been tested yet whether the Rényi entropy allows the adequate modeling of biological phenomena like residue substitutions.

### Global and local methods of correlation analysis complement each other

One goal in the design of H2r, which is a local method, was the identification of individual residue positions important for protein function or stability. This is why we introduced the *conn*(*k*)-value. For two enzymes it has been shown that positions with high *conn*(*k*)-values have an increased probability of being important for enzyme function or stability [36]. The results presented here further confirm the high specificity to be gained with local methods, which is in agreement with data from the literature; see e.g. refs. [19,73]. The results obtained for smHK emphasize that not all correlated mutations are due to functional constraints: 4 of 10 residues with high *conz*(*k*)-values were located at the domain interface and two of them (C217, C224) belong to a disulfide bond that interlinks the domains in some of the homologous proteins. The other residue combinations observed at these two positions illustrate nicely that they were to a great extent occupied by unique residue pairs. Moreover, these findings emphasize a limitation of the *in silico* testbed. Structurally important residues often lay far apart from the catalytic center [74]. As shown above, some bear a strong correlation signal and are identified by H2rs. However, these hits are regarded as false positives and deteriorate the performance values deduced from the testbed.

Whereas local methods consider transitive correlations as well, global methods aim at eliminating these dependencies. The outcome of H2rs and the *L*/5 predictions of the global method PSICOV overlapped only for 22 of 53 residue positions; see Table 2. This result can be explained by the scope of the methods: According to the desired function, global methods identify contacting residue pairs which are not necessarily enriched near functional sites.

Using the MSA *smHK_CMA*, H2rs predicted only three residues known to be functionally important, albeit two with low *conz*(*k*)-values. Using the same dataset, the algorithm Comulator, which aims at identifying perturbations [16], detected a network of six residue positions that surround the active site. Their occupancy almost perfectly separated the two main groups of glucokinases [19]. In summary, these findings highlight the pros and cons of the different approaches and suggest that they supplement each other quite well.

### MSAs have to be prepared carefully

A critical parameter of correlation analysis is the preparation of the input, i.e. the MSA. For the prediction of intra-protein residue contacts, a strong correlation between the number of homologs and the prediction strength has been shown, which further increased, if orthologs and paralogs were included in the MSA [25]. For the sake of standardization, we used in all cases studied here the same methods of MSA preparation without human intervention. Additionally we chose identical and very rigorous cut-offs for the identification of important residue positions. This rigid protocol might be the reason for the considerably differing number of predictions: Using the cut-off *conz*(*k*) ≥ 2.0 and a *p*-values of 10^-11^, H2rs predicted for stTrpA only 2, but for stTrpB 13 important residue positions. These differences suggest for the user an individual adjustment of the parameters for each protein family in order to optimize the benefit of correlation analysis.

## Conclusions

The various global and local methods of correlation analysis have their own field of application and supplement each other. We made plausible that residues in the vicinity of functional sites, which are a large portion of H2rs predictions, do not necessarily belong to residue pairs with the strongest global correlation signal. The predictions of global methods can be assessed by the 3D distance of the involved residue pairs. In contrast, the evaluation of local methods is more ambiguous. Due to the lack of a precise definition of a correlated mutation, it is difficult to specify positive cases. This circumstance has drastic consequences and imposes restrictions to the design and the evaluation of algorithms. With this in mind, we developed an algorithm that considers pairwise substitution frequencies and assesses the strength of the correlation signal statistically. We made plausible that *in silico* testbeds only allow for a rough performance evaluation. Favorable is the detailed analysis of well characterized model systems, which is only feasible for a small number of cases.

## Methods

### Similarity of amino acid pairs and density matrices

Our approach requires for the assessment of two amino acid pairs *i* = (*aa*_*r*_*, aa*_*s*_) and *j* = (*aa*_*t*_*, aa*_*u*_) a similarity matrix **A** of size 400 × 400 such that each entry *a*_*i,j*_ gives a normalized measure for the similarity of the two pairs. To create **A**, we utilized a precompiled and redundancy free list of 35 809 PDB entries [43] offered by the PISCES server [44]. For each protein structure, we analyzed the corresponding MSA from the HSSP database [45]. These MSAs were further processed to eliminate unrelated sequences and closely related ones, which is known to improve the quality of the predictions [25]. This is why we compared for each MSA all pairs of sequences *s*_*r*_, *s*_*s*_ and eliminated sequences *s*_*s*_ until all sequences contained in pairwise comparison at least 20% and not more than 90% identical residues.

Next, we determined for each protein all pairs of residue positions *k*, *l* which are close in 3D space. Distances were determined by using the BALL software library [75] and the cut-off was a maximal distance of 5.0 Å between the centers of any two heavy atoms belonging to one of the corresponding residues. Alternatively a cut-off of 3.5 Å was used. Contacting residues were mapped to the corresponding MSA columns and pairwise amino acid transitions were counted for all sequence pairs to determine substitution frequencies *f*(*i, j*). We adapted a concept, which was introduced for the determination of the BLOSUM matrices [41]; see Figure 1. Each matrix element *a*_*i,j*_ was normalized [38]:

(11) $$a_{i,j}=\frac{f\left(i,j\right)}{\sqrt{f\left(i,i\right)f\left(j,j\right)}}$$

The result is a positive semi-definite similarity matrix **A** with *a*_*i,i*_ = 1 and 0 ≤ a_*i,j*_ ≤ 1 (*i* ≠ *j*) elsewhere. **A** can then be used to calculate density matrices **ρ**_*k*,*l*_ for residue positions *k* and *l*, see Formula (4). The matrix **ρ**_*k,l*_ fulfills all requirements of being a density matrix: First, **ρ**_*k,l*_ is positive semi-definite since **A** is positive definite. Second, by the cyclicity of the trace, the trace of **ρ**_*k,l*_ equals the sum of all probabilities, which is 1 due to our normalization.

### A *p-*value for the significance of pairwise correlations

In order to determine the statistical significance of correlations, we utilized a randomization test and shuffled the columns of the MSA. Consequently, the entropy at each individual position was unchanged, but the correlation between pairs of positions was randomized. Subsequently, we re-calculated a distribution *X* of *U*_*vNE*_ values *x* and repeated this process 25 times, which was sufficient to estimate the mean $\bar{x}$ and the standard deviation σ of *X* needed to approximate a Gumbel distribution [48]. The cumulative Gumbel distribution *F* has the form

(12) $$F\left(x,\mu ,\beta \right)=e^{-e^{-\left(x-\mu \right)/\beta }}$$

and requires two parameters

(13) $$\beta =\frac{\left(\sigma \;\sqrt{6}\right)}{\pi }$$

(14) $$\mu =\bar{x}+\gamma \beta$$

*β* and *μ* result from $\bar{x}$ and *σ* of *X* and *γ* is the Euler–Mascheroni constant (≈0.5772). Using *F*(.), we determined a Bonferroni corrected *p*-value; see Formula (10).

### Characterization of individual residues

In analogy to H2r, H2rs calculates a *conn*(*k*)-value by counting the occurrence of each residue *k* in the set of all significantly correlated pairs of residues. Furthermore, the *conn*(*k*)-values are transformed into z-scores *conz(k)* by

(15) $$\mathrm{conz}\left(k\right)=\frac{\mathrm{conn}\left(k\right)-\bar{\mathrm{conn}\left(k\right)}\;}{\sigma_{\mathrm{conn}\left(k\right)}}$$

where $\bar{\mathrm{conn}\left(k\right)}\;$ and σ_*conn*(*k*)_ are the mean and standard deviation of the distribution of all *conn*(*k*)-values > 0 determined for the protein under study.

### *In silico* testbed and assessment of performance

To statistically evaluate algorithms, we utilized parts of the datasets *CAT_sites* and *LIG_sites* consisting of known catalytic and ligand binding sites, which we have introduced recently [76]. In short, the dataset consists of 200 non redundant PDB entries with corresponding HSSP MSAs [45], each containing at least 125 sequences. Functional sites were identified by means of annotations from the literature entries of the catalytic site atlas [77] and binding site annotations from the PDBsum database [1]. All residues within a maximal distance of 1 Å to a functional site were taken as positives, all other residues as negatives. Subsequently, we determined specificity, and precision:

(16) $$\text{Specificity}=\frac{\text{TN}}{\mathrm{TN}+\mathrm{FP}}$$

(17) $$\text{Precision}=\frac{\mathrm{TP}}{\mathrm{TP}+\mathrm{FP}}$$

In both Formulae, *TP* is the number of true positives, *TN* the number of true negatives, *FP* the number of false positives, and *FN* the number of false negatives.

## Competing interests

The authors declare that they have no competing interests.

## Authors’ contributions

JOJ: Implemented and validated the algorithm H2rs and wrote a first draft of the manuscript. AP deduced the matrix **A**. JB implemented and assessed the algorithm for the computation of the *p*-value. MB was involved in implementing the testbed and determined the performance of CMAT and SCA. MK, WS, and JK designed and assessed the method to compute the *U*_*vNE*_(*k, l*)-values. RM conceived of and managed the project and wrote the final version of the manuscript. All authors read and approved the final version.

## Supplementary Material

Additional file 1 **Similarity Matrix A.** Format Excel. The file contains raw substitution frequencies and normalized values. Click here for file
